# Multi-Omics Analysis Reveals a Dependent Relationship Between Rumen Bacteria and Diet of Grass- and Grain-Fed Yaks

**DOI:** 10.3389/fmicb.2021.642959

**Published:** 2021-08-06

**Authors:** Chenchen Xu, Wenwen Liu, Baozhong Sun, Songshan Zhang, Shou Zhang, Yuanli Yang, Yuanhua Lei, Lan Chang, Peng Xie, Huayi Suo

**Affiliations:** ^1^Institute of Animal Sciences, Chinese Academy of Agricultural Sciences, Beijing, China; ^2^College of Food Science, Southwest University, Chongqing, China; ^3^College of Agriculture and Animal Husbandry, Qinghai University, Xining, China

**Keywords:** yak, rumen, 16S rRNA, metabolomics, bacteria, bacterial diversity

## Abstract

Current information on the differences between rumen bacteria and metabolites of the grass-fed and grain-fed yaks is limited. Understanding the composition and alterations of rumen microbial metabolites is important to clarify its potential role in grass-fed and grain-fed systems. The aim of this research was to explore the influence of different production systems on the functional attributes and metabolites in the rumen microbiota of yak using genomics (Illumina MiSeq sequencing of the 16S rRNA gene) and untargeted metabolomics (UHPLC-QTOF-MS). Rumen samples were obtained from grass-fed (C), grain-fed for 3-month (G3), and grain-fed for 6-month yaks (G6). Results showed that the grain-fed yaks presented a lower rumen bacterial richness and diversity when compared to grass-fed yaks. Bacteroidota, Firmicutes, and Fibrobacterota were the main bacterial phyla. At the phylum and genus level, the grass-fed yaks significantly increased the abundance of Fibrobacterota and Fibrobacter (*p* < 0.05), respectively. The metabolomics analysis revealed that the metabolite profiles differed among the three groups. Compared with the grass-fed group, grain feeding significantly increased azelaic acid, hypoxanthine, uridine, L-phenylalanine, anserine, and decreased alpha-linolenic acid, adenine. Pathway enrichment analysis showed significant differences in metabolic pathways among all comparison groups, but the glycerophospholipid metabolism and alpha-linolenic acid metabolism pathway were common key metabolic pathways. This study showed that the combined analysis of microbiota and metabolites could distinguish different production systems and the fattening time of yaks, providing novel insights for us to understand the function of the rumen bacteria.

## Introduction

The yak (*Bos grunniens*) is the most valuable herbivorous livestock in the Qinghai-Tibetan Plateau, providing necessities like milk, meat, and transportation for local people (An et al., 2005). However, yaks are mainly grazing, and their living environment is challenged by extreme environments such as low temperature, hypoxia, high altitude, and long-term shortage of grain feed, which limits the quality and supply of diet, especially in winter (Ren et al., 2020). To ensure the sustainability of grassland utilization and the growth performance of yak, it is necessary to use management strategies, for instance, supplementary feeding and indoor feeding. In the course of evolution, grass-fed yaks have developed unique genetic traits that determine their ability to adapt to the environment (Qiu et al., 2012).

The rumen is a stable and incredibly complicated micro-ecosystem with a complex microbial community, including countless bacteria, archaea, fungi, and protozoa (Russell and Rychlik, 2001; Qiu et al., 2020). It is well known that the composition of the rumen bacteria is affected by feed diet or feeding methods, which is also the main reason for different feed conversion efficiency for ruminants (Henderson et al., 2015; Zhang et al., 2017; Zhou J. W. et al., 2017). Although previous studies have shown that grain-based feeding cattle had lower bacterial diversity compared with pasture-rich diets (Fernando et al., 2010; Plaizier et al., 2017; Liu et al., 2019), little is known about the interaction of microbial flora and feeding time, in the case of different feed types.

The application of high-throughput sequencing (via 16S rRNA sequencing technology) and metabolomics to analyze the changes of rumen microbiota under different diets is worthy of in-depth exploration. Zhou Z. et al. (2017) investigated the microbial composition of the rumen through high-throughput sequencing. Liu et al. (2019) used high-throughput sequencing and metabolomics to investigate the impacts of different feeding methods on the microbiota and metabolites in the rumen, but the rumen adaptation period was only 15 days. However, the stabilization of the bacterial community in the rumen takes at least 3 months (Qiu et al., 2019). Currently, there is limited information on the differences in microbial diversity and metabolic mechanism between grass-fed and grain-fed yak rumen, after a stable period to adapt to dietary changes.

Therefore, in this study, we combined ruminal microbiome and microbial metabolome to compare the bacterial community diversity and metabolite composition in the rumen of yak fed with grass, grain for 3 months, and grain for 6 months, and determine the potential biomarkers in the rumen fluid under different production systems.

## Materials and Methods

### Animals, Diets, and Trial Design

All animal-specific procedures were approved and authorized by the Animal Ethics Committee of the Chinese Academy of Agricultural Sciences. In this study, the animals were born, raised, and maintained in the Xiahua Beef Cattle Specialized Cooperative Base in Haiyan County, Haibei Tibetan Autonomous Prefecture, Qinghai Province, China. A total of eighteen healthy male yaks (3 years old, bodyweight 150 ± 10.5 kg) were selected and randomly divided into three groups, all of which came from a mixed pasture dominated by carex qinghaiensis and kobresia pygmaea. Grass-fed yaks continued to be reared for 6 months as a control (C, *n* = 6) on a natural pasture. Grain-fed yaks were divided into two groups: grain-fed for 3 months (G3, *n* = 6) and grain-fed for 6 months (G6, *n* = 6) with 40% concentrate and 60% forage. Among them, the yaks in the G3 group lived on pastures for 3 months before being kept in feedlot pens for 3 months. After being selected from the pasture, the yaks of the G6 group were housed in the feedlot pens. The proportions and chemical compositions of the basal diet is shown in Supplementary Table 1.

### Rumen Fluid Sampling

After being weighed (190.7 ± 20.6 kg for the C group, 197.7 ± 17.2 kg for the G3 group, and 228.3 ± 23.1 kg for the G6 group), the yaks were transported to the commercial slaughterhouse. Then, the yaks were stunned by electricity before being slaughtered. Approximately 50 mL of rumen content was collected, and quickly frozen in liquid nitrogen, before processing (sequencing and metabolomics).

### DNA Extraction and High-Throughput Sequencing

The DNA was extracted from ruminal fluid samples using the FastDNA SPIN kit (MP Biomedical, Solon, OH, United States) as instructed by the manufacturer. TBS-380 and NanoDrop2000 were used to determine the concentration and purity of extracted DNA, respectively. The quality of the DNA extract was detected through a 1% agarose gel. Covaris M220 (Gene Company Limited, China) was used to fragment the DNA extract to an average size of about 400 bp for the construction of a paired-end library. The following primers were used to amplify the V3–V4 region of the 16S rRNA gene: 338F, 5′-ACTCCTACGGGAGGCAGCAG-3′ and 806R, 5′-GGACTACHVGGGTWTCTAAT-3′. PCR was conducted using an S1000 thermal cycler (Bio-Rad, Hercules, CA, United States). The extracted PCR amplification products were purified and quantified using AxyPrep DNA Gel Extraction Kit (Axygen Biosciences, Union City, CA, United States) and QuantiFluor-ST (Promega, WI, United States). The sequencing library was constructed with the TruSeq DNA Sample Preparation Kit (Bioo Scientific, Austin, TX, United States), which was completed by Majorbio Bio-Pharm Technology Co., Ltd. (Shanghai, China). The purified amplicons were pooled in equimolar and sequenced on a MiSeq Platform (Illumina, San Diego, CA, United States) using sequencing 2 × 300 bp paired end with the MiSeq^®^ Reagent Kit v3 reagent kit at Majorbio Bio-Pharm Technology Co., Ltd. (Shanghai, China).

### Bioinformatic Analysis

The paired-end reads were trimmed and merged using trimmomatic version 0.32. After removing low-quality sequences and chimeras, the sequences were clustered into Operational Taxonomic Units (OTUs) with 97% sequence similarity. The classification of each 16S rRNA gene sequence database (Silva version 138) was analyzed with a 70% reliability threshold (Quast et al., 2012). The rarefaction curves and bar graphs used to analyze the abundance of bacteria in all samples were made by the vegan package in R (Hsieh et al., 2016). Alpha diversity indices were performed with Mothur (version v.1.30.2). The results of principal coordinate analysis (PCoA) were performed with GUnifrac and ape packages in R (Lozupone and Knight, 2005; Mao et al., 2016). Venn diagrams illustrating the overlap of OTUs between groups were generated in R (1.6.20) (Chen, 2018). The difference in the abundance of microbial communities between samples obtained by statistical methods was used to assess the significance of the difference using FDR (false discovery rate). The *p*-value obtained by adjusting the false discovery rate (FDR) by the Benjamini–Hochberg method. *p* < 0.05 was shown as statistically significant in the relative abundance of microbiota among the three feeding methods.

### Metabolomics Analysis

The thawed rumen fluid samples were filtered through four layers of gauze, and then centrifuged at 11,200 × *g* for 10 min. The supernatant (100 μL) of each sample was mixed with 10 μL of internal standard (3.0 mg/mL, L-2-chlorophenylalanine), and methanol-acetonitrile (300 μL, 2:1, v/v), and then vortexed for 1 min. The LC-MS/MS analyses were carried out with an Agilent 1290 Infinity LC system (Agilent Technologies, Santa Clara, CA, United States) with an Acquity BEH C18 column (100 mm × 2.1 mm i.d., 1.7 μm, Waters, Milford, MA, United States) preheated to 45°C. The mobile phase was composed of solvent A (aqueous 0.1% (v/v) formic acid) and solvent B (acetonitrile) delivered at 0.40 mL/min with the following gradient: 0–1 min, 1% B, 1–5.5 min, 1% B–20% B, 5.5–6 min, 20% B–30% B, 6–8.5 min, 30% B–35% B, 8.5–10.5 min, 35% B–70% B, 10.5–11 min, 70% B–100% B, 11–13 min, 100% B, 13–13.1 min, 100% B–1% B, and 13.1–15 min, 1% B. The injection volume was 3 μL. The LTQ Orbitrap mass spectrometer (XL, Thermo Fisher Scientific, Waltham, MA, United States) was used to obtain sample mass spectrum data using positive ion or negative ion scan mode. The electron spray ionization source conditions were set as follows: sample voltage, 40 V, capillary voltage, 1.0 kV, ion source temperature 120°C, desolvation gas rate and temperature, 900 L/h and 500°C, the mass range, 50–1000 m/z, scan time and interscan delay, 0.15 and 0.02 s, respectively. The normalized collision energy was 6 eV.

### Data Analysis

The preprocessing included the integration of peak intensities, normalization and peak alignment, and produced a data matrix composed of retention time (RT), the mass-to-charge ratio (m/z) values, and peak intensity finally. The processed data were input to the SIMCA-P software (V14.0, Umetric, Umeå, Sweden) using principal component analysis (PCA) and orthogonal partial least-squares discriminate analysis (OPLS-DA) to detect metabolic changes. Each metabolite received a value called variable importance for the projection (VIP). Differentially expressed metabolites (DEMs) were screened using Student’s *t*-test. If *p* < 0.05 and VIP > 1, the variables were considered to be significantly different in metabolites between the two groups. Enrichment analysis and pathway topology analysis were acquired by using MetaboAnalyst 4.0^1^ and Kyoto Encyclopedia of Genes and Genomes (KEGG).^2^ The functional correlation between metabolites and rumen bacterial changes was conducted using Spearman’s correlation.

## Results

### 16S rRNA Gene Sequencing Results

A total of 906,639 sequences (307,480 reads from C group, 291,459 reads from G3 group, and 307,700 reads from G6 group) with an average length of 441 bp were obtained through the 16S rRNA gene sequencing. Our study also obtained 5,285 OTUs at a sequence-similarity level of 97%. In Supplementary Figure 1, the Venn figure demonstrated that there were 1,178 common OTUs among the three groups. The rarefaction curves of each sample reached the plateau level (Supplementary Figure 2), indicating that the sequencing depth was sufficient.

### Bacterial Diversity Analysis

**FIGURE 1:**
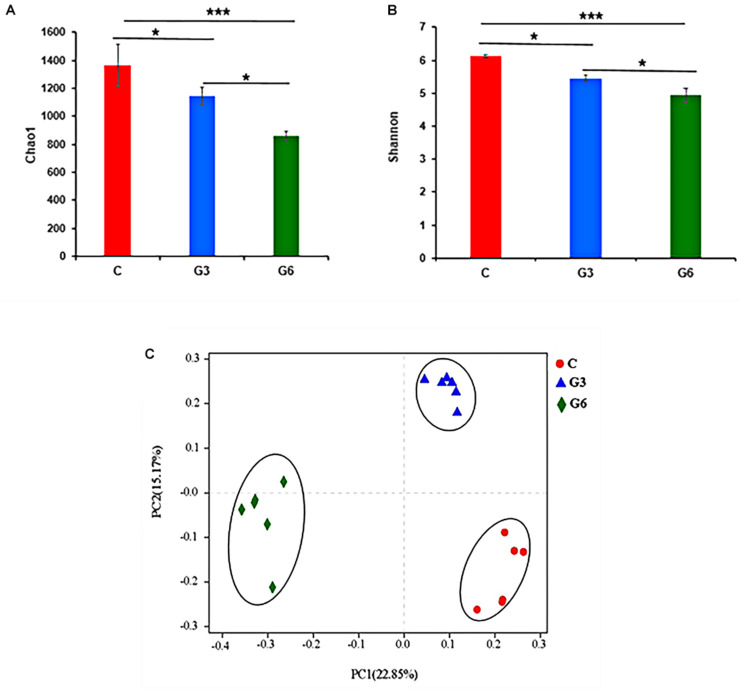
Bacterial diversity. **(A,B)** Alpha diversity metrics **(A)** Chao 1 and **(B)** Shannon for the rumen microbiota of grass-fed (C), grain-fed for 3 months (G3), and grain-fed for 6 months (G6) yaks. **(C)** The plot of Principal coordinate analysis (PCoA). The closer distance between the sample points means the higher similarity of the bacteria. *p < 0.05; ***p < 0.001.

Alpha diversity indices, including Chao1 and Shannon, are shown in Figures 1A,B. The Chao1 and Shannon indices in the grass-fed group (C) had higher values than the grain-fed group (G3 and G6) (*p* < 0.05), suggesting that the microbial diversity of the grass-fed group is higher than that of the grain-fed group. The results of beta diversity did show a significant difference among the three groups. PCoA showed the difference of microbiota in the samples of grass-fed, grain-fed for 3 months, and grain-fed for 6 months yak rumen (Figure 1C). The plot demonstrated that the microbial differentiation of the three groups had a clear separation, indicating that the microbial communities had obvious differences.

### Rumen Microbial Composition at the Phylum and Genus Levels

**FIGURE 2:**
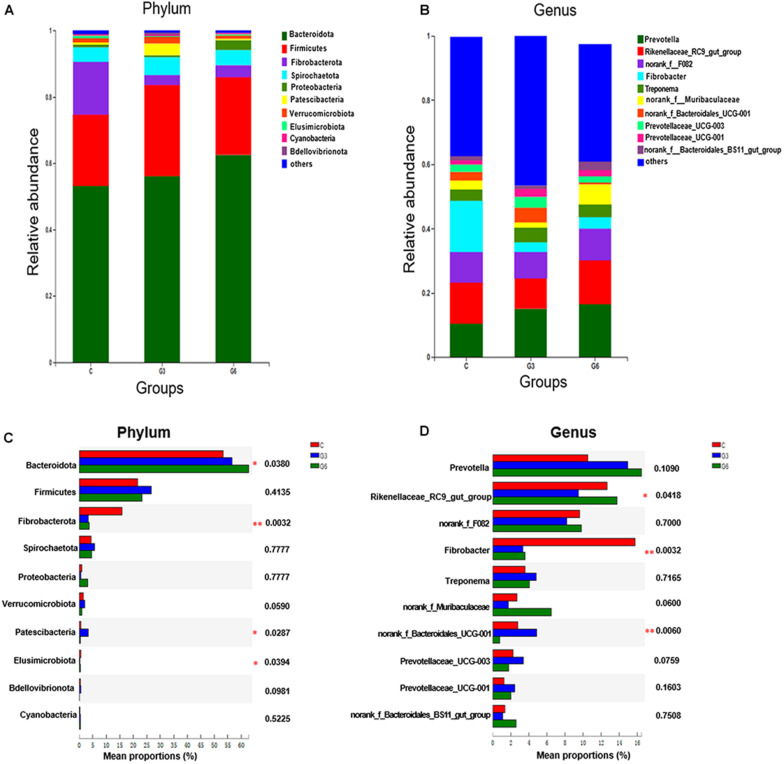
Relative rumen microbiota abundance at **(A,C)** phylum and **(B,D)** genus levels in the grass-fed (C), grain-fed for 3 months (G3), and grain-fed for 6 months (G6) yaks experimental groups. *p < 0.05; **p < 0.01.

Figure 2 shows the differences in relative abundances of the top 10 most abundant microbial species in three groups. The results demonstrated that Bacteroidota, Firmicutes, and Fibrobacterota were the main bacterial phyla (Figure 2A). Bacteroidota and Firmicutes accounted for 53.22 and 21.46% in the C group, and 56.15 and 27.43% in the G3 group, and 62.51 and 23.46% in the G6 groups, respectively. Compared with the C group, Bacteroidota increased (*p* < 0.05) in the G6 group. In grass-fed yaks, the relative abundance of Fibrobacterota (15.88%) was significantly higher (*p* < 0.05) than that in grain-fed yaks (G3, 3.05% and G6, 3.60%). However, the relative abundances of the Patescibacteria in the G3 group was higher (*p* < 0.05) than that in C and G6 groups (Figure 2C). At the genus level, *Prevotella*, *Rikenellaceae_RC9_gut_group*, and *norank_f_F082* were the main genera in these three groups (Figure 2B). Compared with the G6, the *Rikenellaceae_RC9_gut_group* of the G3 was significantly reduced (*p* < 0.05). While the relative abundance of Fibrobacter showed the highest value (*p* < 0.01) in C group, and *norank_f_Bacteroidales_UCG-001* showed the lowest value (*p* < 0.01) in G6 group (*p* < 0.05), respectively. No significant differences were found in *Prevotella*, *norank_f_Bacteroidales_BS11_gut_group*, *norank_f_F082*, *Treponema, norank_f_Muribaculaceae*, *Prevotellaceae_UCG-003*, and *Prevotellaceae_UCG-001* (*p* > 0.05) among three groups (Figure 2D).

### Metabolomic Profiles of the Rumen Fluid

**FIGURE 3:**
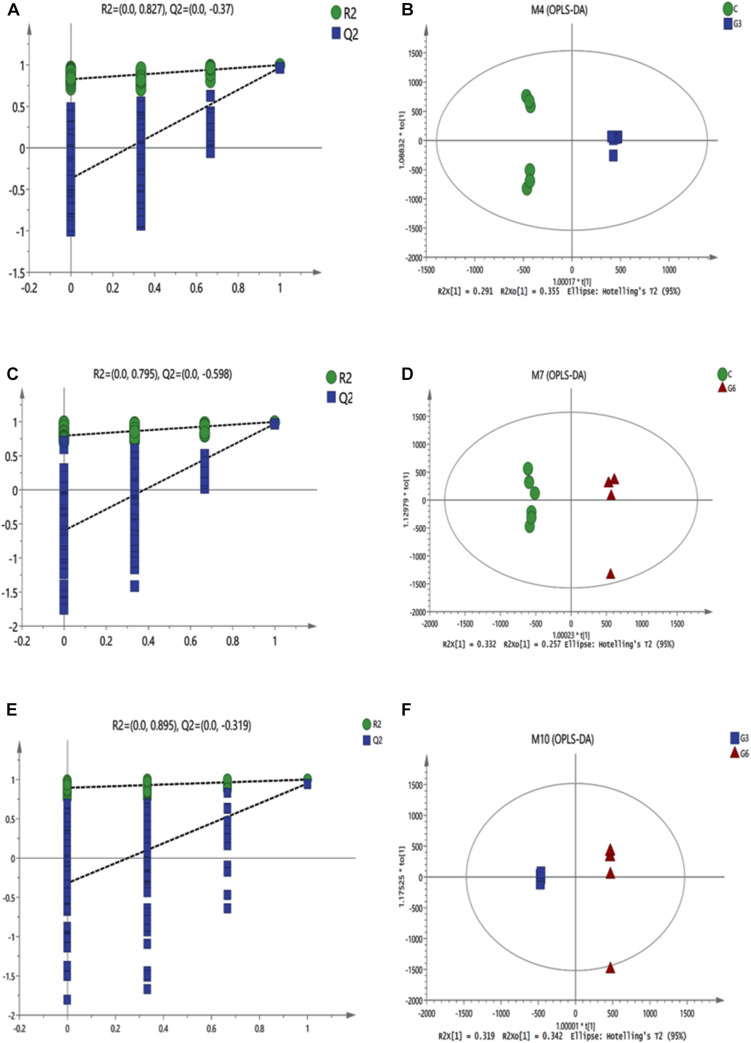
The permutation test (*n* = 200) of the OPLS-DA model **(A,C,E)** and OPLS-DA model **(B,D,F)** were derived from the liquid chromatography/mass spectrometry metabolomics profiles of yak rumen fluid samples from grass-fed (C), grain-fed for 3 months (G3), and grain-fed for 6 months (G6) groups. Green and blue respectively represent R2 value and Q2 value from the permutation test **(A,C,E)**. The green circles represent the C group, blue squares represent the G3 group, and the red triangles represent the G6 group **(B,D,F)**.

In this research, the PCA score plot confirmed the validity of the data (Supplementary Figure 3). Figure 3 showed the contribution of different variables to the discriminating ability of each OPLS-DA model. As shown in Figure 3A, the permutation test (C versus G3, R^2^Y = 0.827, Q^2^Y = −0.37 < 0, C versus G6, R^2^Y = 0.795, Q^2^Y = −0.598 < 0, G3 versus G6, R^2^Y = 0.895, Q^2^Y = −0.319 < 0) suggested that the model was adequate for its efficacy (Figures 3A,C,E). Except for 1 rumen fluid sample outside the ellipse in the score plots, the other samples were all inside the 95% Hotelling T2 ellipse (Figures 3B,D,F). All data in the three models in the C versus G3 groups, C versus G6 groups, and G3 versus G6 groups showed significant separation and differentiation, illustrating the effectiveness of the OPLS-DA model in a further screening of different metabolites.

### Identification of Metabolites

Overall 1,950 metabolites were identified. Using the criteria VIP > 1 and *p* < 0.05, 264 DEMs were detected in Group C versus Group G3, 261 DEMs were detected in Group C versus Group G6, and 271 DEMs were detected in Group G3 versus Group G6 (Supplementary Table 2). These different metabolites are mainly lipids, carbohydrates, organic acids, nucleic acids, peptides, steroids, hormones and transmitters, and vitamins and cofactors. Compared with the C group, azelaic acid, hypoxanthine, uridine, L-phenylalanine, anserine in the G3 group and G6 group were increased, and alpha-linolenic acid, and adenine were reduced in G3 and G6 groups (Table 1). Compared with the yak fed with grain for 3 months, the metabolites of organic acids, lipids, and peptides in the rumen fluid were upregulated after feeding grain for 6 months. Inversely, the concentrations of some nucleic acid metabolites such as thymidine, hypoxanthine, and uridine 5’-monophosphate, showed a significant decrease.

**TABLE 1:** Potential distinguishing metabolites in the rumen fluid between three groups.

**Groups^1^**	**Metabolites**	**VIP^2^**	***p*-value**	**Fold Change**	**Type**	**Metabolic classes**
C vs G3	Azelaic acid	4.84917	0.017	1.533	Up	Organic acids
	Suberic acid	1.5952	0.015	0.461	Down	
	Eicosadienoic acid	1.36396	0.000	3.891	Up	Lipids
	Alpha-Linolenic acid	1.24425	0.032	0.691	Down	
	Linoleic acid	3.10554	0.008	1.197	Up	
	Undecanedioic acid	1.95382	0.002	1.334	Up	
	Cytosine	1.10418	0.000	3.036	Up	Nucleic acids
	Hypoxanthine	2.26807	0.040	1.258	Up	
	Adenine	4.50394	0.021	0.435	Down	
	Uridine	2.08764	0.001	2.165	Up	
	Adenosine monophosphate	2.39806	0.016	3.344	Up	
	Uridine 5′-monophosphate	4.55688	0.000	5.251	Up	
	Guanosine monophosphate	1.6309	0.005	5.064	Up	
	Homocysteine	1.36719	0.035	0.017	Down	Peptides
	L-Phenylalanine	1.54323	0.012	1.552	Up	
	Anserine	1.59658	0.000	9.053	Up	
	Melatonin	2.45201	0.031	2.407	Up	Hormones and transmitters
C vs G6	D-Glucose	1.15468	0.045	0.366	Down	Carbohydrates
	Azelaic acid	7.53028	0.000	2.538	Up	Organic acids
	Undecanedioic acid	2.35238	0.001	1.726	Up	Lipids
	Alpha-Linolenic acid	1.50393	0.001	0.422	Down	
	Adenine	3.53659	0.024	0.360	Down	Nucleic acids
	Hypoxanthine	2.26807	0.040	1.258	Up	
	Uridine	1.37604	0.000	1.810	Up	
	L-Tyrosine	1.23263	0.005	1.415	Up	Peptides
	L-Phenylalanine	1.57922	0.000	1.773	Up	
	Anserine	2.28498	0.001	32.024	Up	
	Chenodeoxycholic acid	1.96605	0.022	0.925	Down	Steroids
G3 vs G6	Azelaic acid	1.00161	0.003	1.655	Up	Organic acids
	Suberic acid	1.4799	0.004	2.611	Up	
	Undecanedioic acid	1.86879	0.015	1.294	Up	Lipids
	Alpha-Linolenic acid	1.13448	0.005	1.936	Up	
	Thymidine	1.88592	0.000	0.661	Down	Nucleic acids
	Hypoxanthine	3.07616	0.000	0.828	Down	
	Uridine 5′-monophosphate	3.00574	0.000	0.517	Down	
	L-Tyrosine	1.18014	0.043	1.318	Up	Peptides
	L-Proline	1.15698	0.000	1.997	Up	
	Anserine	2.03048	0.000	3.538	Up	
	Chenodeoxycholic acid	2.25819	0.000	0.940	Down	Steroids
	Nicotinic acid	2.61928	0.000	1.457	Up	Vitamins and cofactors

*^1^C, grass-fed yaks; G3, grain-fed for 3 months yaks; G6, grain-fed for 6 months yaks.*

*^2^VIP, variable importance in the projection.*

*All different metabolites listed here are those VIP > 1 and *p*-value < 0.05.*

### KEGG Annotation and Metabolic Pathway Analysis of Differential Metabolites

**FIGURE 4:**
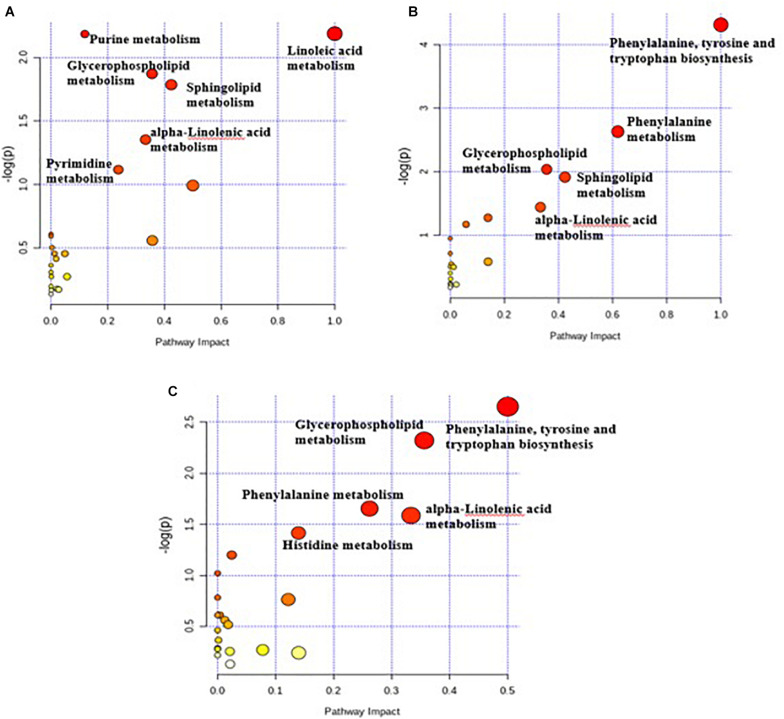
Pathway impact resulting from the differential ruminal metabolites based on *Bos taurus* KEGG database using MetaboAnalyst 4.0. The X-axis represents pathway impact and the Y-axis represents the pathway enrichment. The larger size of the circle indicates greater pathway enrichment and the darker color indicates higher pathway impact values. The closer the color is to red, the smaller the *p*-value is. **(A)** Metabolome view map of metabolites between C and G3 yaks; **(B)** metabolome view map of metabolites between C and G6 yaks; **(C)** metabolome view map of metabolites between G3 and G6 yaks.

Pathway topology analysis was carried out according to the metabolites identified and the concentrations. The six enriched major metabolic pathways between the C and G3 have been shown in Figure 4A, including linoleic acid metabolism, purine metabolism, glycerophospholipid metabolism, sphingolipid metabolism, alpha-linolenic acid metabolism, and pyrimidine metabolism. Figure 4B displayed the five enriched metabolic pathways of C versus G6, including phenylalanine, tyrosine, and tryptophan biosynthesis metabolism, phenylalanine metabolism, glycerophospholipid metabolism, sphingolipid metabolism, and alpha-linolenic acid metabolism.

The five important key metabolic pathways of G3 versus G6 included phenylalanine, tyrosine and tryptophan biosynthesis metabolism, glycerophospholipid metabolism, phenylalanine metabolism, alpha-linolenic acid metabolism, and histidine metabolism (Figure 4C). The pathway impact values of these metabolic pathways were higher than 0.1, which is the critical value for relevance. Although the highly important enrichment metabolic pathways (*p* < 0.01) of the three comparison groups were different, the highly important enrichment metabolic pathways of all comparison groups include glycerophospholipid metabolism and alpha-linolenic acid metabolism.

### The Relationship Between the Metabolites and Rumen Bacteria

**FIGURE 5:**
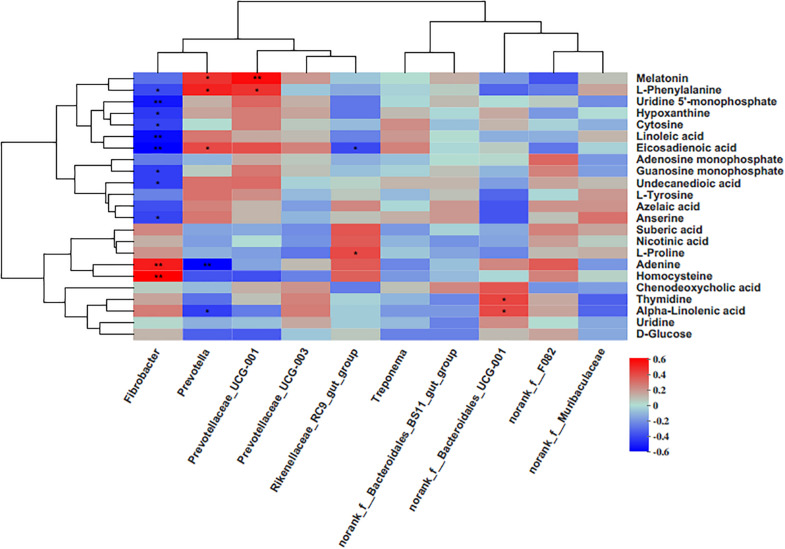
Correlation matrix between the genera level and the ruminal differential metabolites (used in Table 1). Each column in the graph represents a genus, each row represents a metabolite, and each lattice represents a Pearson correlation coefficient between a metabolite and a component. Red represents a positive correlation, while blue represents a negative correlation. *p < 0.05; **p < 0.01.

The results of this study showed that the genus Fibrobacter was positively associated with homocysteine and adenine, while negatively correlated with anserine, undecanedioic acid, guanosine monophosphate, eicosadienoic acid, hypoxanthine, uridine 5′-monophosphate, and L-phenylalanine (Figure 5). The genus *Prevotella*, having a higher abundance in the grain-fed groups (G3 and G6), was positively associated with eicosadienoic acid, melatonin and L-phenylalanine, and negatively correlated with adenine, and alpha-linolenic acid. The genus *Prevotellaceae_UCG-001* was positively correlated with melatonin and L-phenylalanine. *Rikenellaceae_RC9_gut_group* was positively correlated with L-proline, and negatively correlated with eicosadienoic acid. There was a higher level of *norank_f_Bacteroidales_UCG-001* in the G3 group, which was positively correlated with thymidine and alpha-linolenic acid.

## Discussion

Considering that diet is directly related to the microbiome, this study explored the influence of diet type and diet time on the rumen microbiome and metabolome, which could further provide a reference for selective breeding of yak by manipulating the relationship between diet and rumen microbiome.

The species diversity and abundance of rumen microbiota play a vital role in maintaining the normal physiology of the host. According to previous studies, 16S rRNA gene sequencing was sufficient to illustrate the bacterial diversity in the foregut of herbivores (Shanks et al., 2011; Jami and Mizrahi, 2012; Wang et al., 2018). In this study, the sequencing results revealed most of the microbiota in the sample. The Chao1 value reflected the bacterial species richness, and the Shannon value reflected the bacterial diversity. Our findings proved that the rumen samples of grass-fed yaks showed both higher richness and diversity when compared to grain-fed yaks, consistently with other reports (Plaizier et al., 2017; Liu et al., 2019; Qiu et al., 2020), indicating that the grain-based diet reduced the richness and diversity of rumen bacteria. The results also clarified that the abundance and diversity of rumen bacteria decreased significantly with the extension of the grain diet. The PCoA analyses displayed obvious clusters, and significant differences were observed in the rumen microbial communities among the three groups.

In the current study, the phyla Bacteroidota and Firmicutes were dominant bacteria in the yak rumen in three groups, in agreement with previous findings in yaks (Zhou Z. et al., 2017); Xue et al., 2018; Hu et al., 2019). The abilities of Bacteroidota and Firmicutes to degrade structural polysaccharides have been previously reported (Zened et al., 2013). The main role of Bacteroides is to improve the nutrient utilization of the host by helping the host degrade carbohydrates and to enhance the host’s immunity (Bäckhed et al., 2005; Sears, 2005). We also found that the abundance of Bacteroidota of the grain-fed yak rumen was significantly higher than that in the grass-fed yak. This finding indicated that Bacteroidetes in the rumen show adaptive changes under long-term grain feeding, mainly involved in the degradation of carbohydrates. Fibrobacterota, as well as Fibrobacter, is known for being an important bacterial phylum for ruminants to degrade and digest plant cellulose (Ransom-Jones et al., 2012), and they are usually detected in fiber-rich diets (Fernando et al., 2010). In this study, the abundance of Fibrobacterota in the C group was much higher than that in the G3 and G6 groups, which indicated that Fibrobacterota might be actively involved in the digestion of fiber. Our data showed that the G3 group harbored greater proportions of Patescibacteria, that is, higher abundances than in the C and G6 groups, which meant that the feed type and the grain-fed time affected the changes in microbia population structure. Differences in microbiota may explain their changes in more detail (Qiu et al., 2019). The relative abundance of *Rikenellaceae_RC9_gut_group* increased with the increase of grain-feeding time, which was similar to the result of a previously reported study (Wang et al., 2019), indicating that this genus may be involved in the carbohydrate degradation.

Principal component analysis and OPLS-DA showed a significant difference in the metabolic components of rumen fluid between the grass-fed and grain-fed groups, and proved the obvious effect of grain-feeding time on rumen metabolites. Many studies have shown that the grain diet could result in the accumulation of organic acids in the rumen (Kleen et al., 2003; Yang et al., 2018a). We observed the same result in our study, as grain feeding increased the concentration of azelaic acid in the rumen when compared to grass feeding. Moreover, compared with the G3 group, the azelaic acid and suberic acid were increased in the G6 group. The superposition of these changes led to a decrease of the rumen pH, which also verified that the richness and diversity of rumen bacteria in the G6 group in this study were lower than the other two groups.

From the perspective of ruminant health, grain diet is prone to cause an acidic intestinal environment, which affects the rumen. The grass-fed method may be a better choice to avoid this phenomenon (Liu et al., 2020). Nucleic acid metabolites such as cytosine, hypoxanthine, uridine, adenosine monophosphate, uridine 5’-monophosphate, and guanosine monophosphate in the rumen were higher in the G3 rumen when compared to C group. This reflected on the degradation of certain bacterial nucleic acids in the rumen due to grain diets, which was supported by the research from McAllan and Smith (1973). They found that bacterial nucleic acid (RNA or DNA) incubated with rumen fluid was quickly converted into xanthine, hypoxanthine, and uracil. However, with the extension of grain-feeding time, the concentration of thymidine, hypoxanthine, and uridine 5′-monophosphate decreased. A possible explanation for this result is that the lower bacterial diversity and abundance in the rumen led to a decrease of bacterial nucleic acid that could be degraded. The study of the pathway topology determined that the main metabolic pathways were purine metabolism and pyrimidine metabolism. In addition, according to the correlation analysis in the present result, the hypoxanthine was negatively associated with Fibrobacter. Therefore, it may be possible to use hypoxanthine as a biomarker to identify grass-fed or grain-fed production systems. The amino acids in the rumen were mainly derived from dietary proteins degraded by the rumen microbiota (Zhang et al., 2019). L-phenylalanine in the G3 and G6 groups was significantly higher than the C group. Moreover, pathway analyses indicated that the most abundant pathways after 6 months of grain feeding include phenylalanine, tyrosine and tryptophan biosynthesis, and phenylalanine metabolism. These two metabolic pathways were related to phenylalanine, illustrating the important role of phenylalanine in the metabolic pathways of grain-fed diets. The production of tyrosine by the catalysis of phenylalanine hydroxylase was the main metabolic pathway of phenylalanine (Yang et al., 2018b). This could also explain that the concentration of tyrosine in the rumen fluid increased after 6 months of grain feeding. Biohydrogenation, active lipolysis, and microbial fatty acid synthesis are indicated by the concentration of long-chain fatty acids in the rumen (Kim et al., 2009). Compared with the C group, alpha-linolenic acid decreased in the G3 and G6 groups. In addition, with the increase of grain-feeding time, the metabolites involved in lipid metabolism decreased. The enriched pathways observed in this study, including linoleic acid metabolism, and alpha-linolenic acid metabolism. Rumen microbiota would rapidly hydrogenate unsaturated fatty acids ingested by the rumen through diet. Before being hydrogenated into a saturated end product, galactosidase, lipase, and phospholipase produced by rumen microbes remove unesterified fatty acids (Jenkins, 1993). This process can produce different intermediate fatty acids, especially odd-chain fatty acids (Jenkins et al., 2015). Therefore, linoleic acid and alpha-linolenic acid may be rumen potential biomarkers.

The rumen microbial composition of grass-fed and grain-fed yak was significantly different. Grain feeding reduced the richness and diversity of microbiota. Long-term feeding of grains could reduce the richness and diversity of microbiota further. These changes in microbiota were related to lipid metabolism, amino acid metabolism, and nucleotide metabolism. The rumen metabolites azelaic acid, suberic acid, hypoxanthine, phenylalanine, linoleic acid, and alpha-linolenic acid had sufficient sensitivity and specificity to distinguish grass-fed and grain-fed yaks. The metabolites may be used as potential biomarkers to predict rumen function in different production systems. Our study contributes to a comprehensive understanding of the dynamic and complex relationships between diet and rumen microbiota function, which is essential for formulating appropriate feeding strategies to improve yak production performance.

## Data Availability Statement

Publicly available datasets were analyzed in this study. This data can be found here: https://www.ncbi.nlm.nih.gov/bioproject/PRJNA657445.

## Ethics Statement

The animal study was reviewed and approved by Animal Ethics Committee of the Chinese Academy of Agricultural Sciences.

## Author Contributions

WL, HS, and SZ designed the study. YY, YL, SZ, and LC carried out the sample processing. CX performed the experimental data analysis and wrote the manuscript. PX and BS modified the manuscript. All authors have read and agreed to the published version of the manuscript.

## Conflict of Interest

The authors declare that the research was conducted in the absence of any commercial or financial relationships that could be construed as a potential conflict of interest.

## Publisher’s Note

All claims expressed in this article are solely those of the authors and do not necessarily represent those of their affiliated organizations, or those of the publisher, the editors and the reviewers. Any product that may be evaluated in this article, or claim that may be made by its manufacturer, is not guaranteed or endorsed by the publisher.

## Footnotes

1
http://www.metaboanalyst.ca

2
http://www.genome.jp/kegg/

## Supplementary Material

Supplementary Figure 1 Venn diagram of Operational Taxonomic Units (OTUs) in three groups.

Supplementary Figure 2 Rarefaction curves for each sample in three groups.

Supplementary Figure 3 Principal compon ent analysis (PCA) score plots of metabolite profile. Ellipses represent the 95% confidence in the score plots. The green circles represent grass-fed yaks (C), the blue squares represent grain-fed for 3 months yaks (G3), the red triangles represent grain-fed for 6 months yaks (G6), and the yellow inverted triangles represent the quality control (QC) samples.

Supplementary Table 1 Ingredients and chemical composition of the formulation.

Supplementary Table 2 List of identified metabolites.
