# Supplementary figures and images for: Multi-Omics Analysis Reveals a Dependent Relationship Between Rumen Bacteria and Diet of Grass- and Grain-Fed Yaks
Source: Front Microbiol. 2021 Aug 6;12:642959. doi: 10.3389/fmicb.2021.642959 (PMC8377600; doi:10.3389/fmicb.2021.642959)

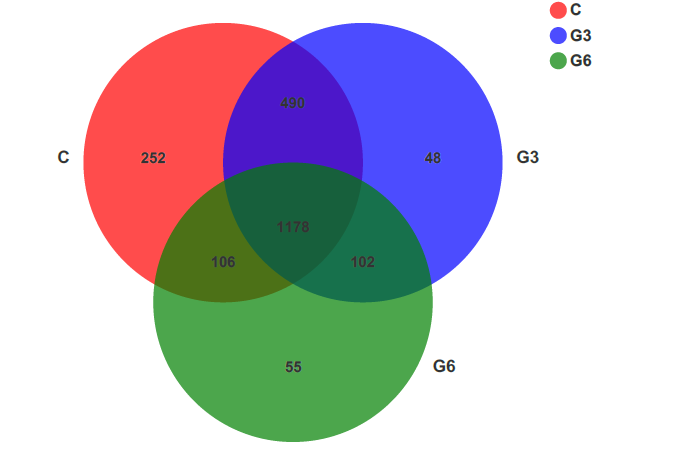

Supplement: Supplementary Figure 1 — Venn diagram of Operational Taxonomic Units (OTUs) in three groups. [file Image_1.PNG]

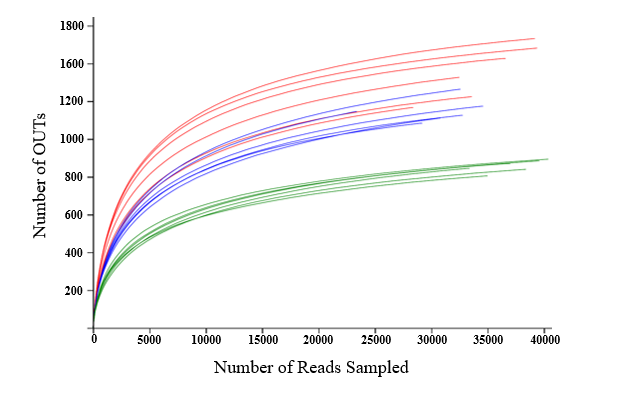

Supplement: Supplementary Figure 2 — Rarefaction curves for each sample in three groups. [file Image_2.PNG]

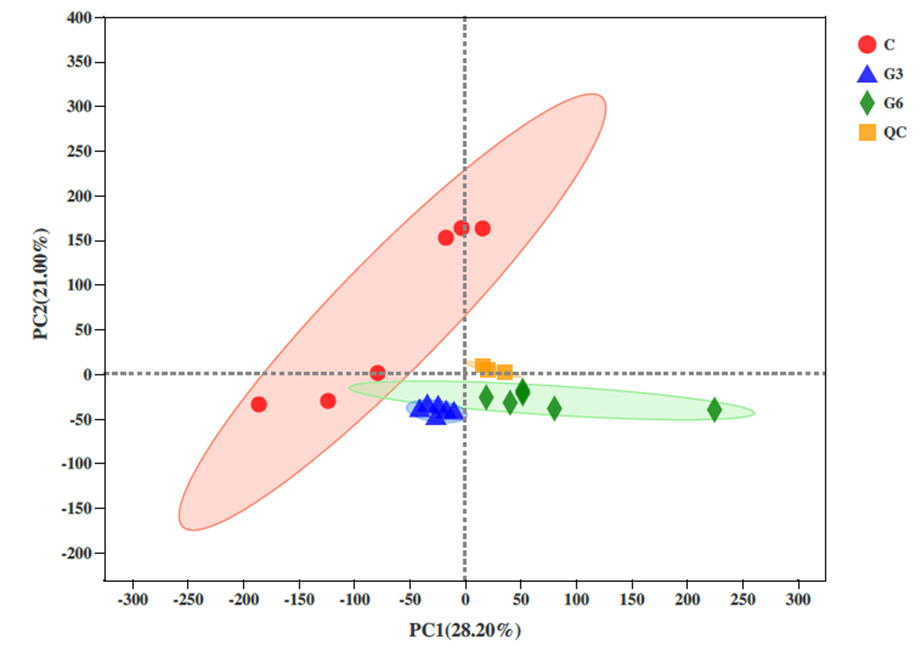

Supplement: Supplementary Figure 3 — Principal compon ent analysis (PCA) score plots of metabolite profile. Ellipses represent the 95% confidence in the score plots. The green circles represent grass-fed yaks (C), the blue squares represent grain-fed for 3 months yaks (G3), the red triangles represent grain-fed for 6 months yaks (G6), and the yellow inverted triangles represent the quality control (QC) samples. [file Image_3.PNG]
