# Supplementary material for: Multi-Omics Analysis Reveals a Dependent Relationship Between Rumen Bacteria and Diet of Grass- and Grain-Fed Yaks
Source: Front Microbiol. 2021 Aug 6;12:642959. doi: 10.3389/fmicb.2021.642959 (PMC8377600; doi:10.3389/fmicb.2021.642959)
Supplement: Supplementary Table 1 — Ingredients and chemical composition of the formulation. [file Table_1.docx]

**Supplementary Table S1**. Ingredients and chemical composition of the formulation

| Items | Ratio (%) |
| --- | --- |
| Ingredients (DM basis) |  |
| Oat silage | 32.5 |
| Oat Hay | 27.5 |
| Corn | 29.6 |
| Soybean meal | 1.4 |
| Corn distillers dried grains with solubles | 2.4 |
| Fermented distiller’s grains | 2.6 |
| Corn fiber | 1.3 |
| Salt | 0.4 |
| Sodium bicarbonate | 0.4 |
| Choline Chloride | 0.1 |
| Limestone | 0.3 |
| Premix^1^ | 1.5 |
| Chemical composition (DM basis) |  |
| NEg^2^ (MJ/kg DM) | 5.1 |
| Crude protein | 12.2 |
| Ether extract | 4.5 |
| Neutral detergent fiber | 31.6 |
| Acid detergent fiber | 16.8 |
| Calcium | 0.4 |
| Phosphorus | 0.3 |

^1^The premix provided the following kg^-1^ diets: VA 5 500 IU, VD_3_ 1 920 IU, VE 20 IU, Fe 50 mg, Cu 10.5 mg, Mn 20.3 mg, Zn 30 mg, I 0.52mg, Se 0.1 mg, Co 0.12 mg.

^2^NEg, net energy for gain, is calculated according to Feeding Standard of Beef Cattle (NY/T 815–2004), others were measured values.
